# Schwannoma and Post-vaccine Changes: A Case Report

**DOI:** 10.7759/cureus.48223

**Published:** 2023-11-03

**Authors:** Martha-Lilia Tena-Suck, Steven-Andrés Piña-Ballantyne, Jesús Cienfuegos-Meza, Marco-Antonio Jiménez-López, Andrea Ávalos-Arias

**Affiliations:** 1 Neuropathology, Instituto Nacional de Neurología y Neurocirugia Manuel Velasco Suárez, Mexico, MEX; 2 Neuropathology, Instituto Nacional de Neurología y Neurocirugía Manuel Velasco Suárez, Mexico, MEX; 3 Pathological Anatomy, Hospital General 450, Durango, MEX; 4 Neurology, Instituto Nacional de Neurología y Neurocirugía Manuel Velasco Suárez, Mexico, MEX

**Keywords:** covid-19 vaccine, hemorrhages and macrophages, necrosis, secondaries changes, cervical schwannoma

## Abstract

Schwannomas are benign sheaths of Schwann cells that can present with degenerative and morphological changes; necrosis or hemorrhage are rare findings in these tumors. We present the case of a 28-year-old man with a C2-C4 cervical Schwannoma who experienced upper limb paresthesia in 2020 while presenting with COVID-19 symptoms. The patient later recovered and came to our institution, where surgery was scheduled one year after the initial diagnosis. One week before surgery, the patient received the first dose of the Moderna vaccine. Despite being asymptomatic, the patient underwent successful total resection of the schwannoma, which was confirmed histologically. However, extensive necrosis with abundant foamy macrophages was observed, suggesting a possible link to post-vaccine effects.

## Introduction

Schwannoma is a benign, slow-growing tumor composed of well-differentiated sheath Schwann cells, with a loss of myelin (NF2 gene product) expression [1]. In the most updated version of the World Health Organization (WHO) classification of tumors of the central nervous system, the term "malignant melanotic nerve sheath tumor," previously called melanotic schwannoma, was changed [1]. Conventional and non-melanocytic subtypes are classified as WHO grade one [1]. Histologically, schwannomas (SCs) are divided into different types: common, plexiform, cellular, epithelioid, and ancient SCs [2]. The complexity of SC pathogenesis arises from the crucial roles of each cell type involved in tumorigenesis in establishing an ideal microenvironment [3]. Degenerative changes like cyst formation, hyalinization, and proliferation of vessels, as well as inflammation, macrophages, xanthomatous changes, and infected SCs with abundant inflammation, have been described by histologists [2,4-7]. These findings are known to occur in SCs and may often make diagnosis difficult [8].

SARS-CoV-2 invades the nervous tissue through angiotensin-converting enzyme 2 (ACE2), transmembrane protease, serine 2 (TMPRSS2), or NRLP-1 (a member of the NOD-like receptors) receptors, which are expressed in several cells, including neurons and glial cells [9]. The virus is detectable by reverse transcriptase polymerase chain reaction (PCR), electron microscopy (virions), or immunohistochemical staining (using SARS-CoV-2 antibodies) [9].

Neuropathology and coronavirus disease (COVID-19) infection have been well characterized, including extensive necrosis with inflammation, thrombosed vessels, fibrinoid necrosis, endothelial damage, and multifocal microvascular injury. Immunohistochemical staining for CoV-2 was positive in perivascular inflammatory and endothelial cells [10,11].

We present a rare case of a young man who presented with COVID-19 six months before and later presented with paresthesia; in the upper limbs, the patient was diagnosed with schwannoma in C2. He was scheduled for surgery at the time of programming and had been vaccinated with the Moderna vaccine a week prior. Histologically, the tumor presents with abundant inflammation, vascular damage, foci of necrosis, fibrinoid necrosis, and newly formed vessels.

## Case presentation

A 28-year-old male with a history of cocaine and alcohol consumption quit three years ago. In February 2019, the patient presented with decreased muscle strength along with ipsilateral numbness of the arms, inability to walk, and hypoesthesia. In March 2021, he started with a sweaty, nocturnal fever of 39-40 degrees Celsius (C) accompanied by diarrhea that lasted a week, during which time he lost 10 kg of weight.

No COVID-19 test was performed at that time. Subsequently, in July 2021, he experienced general malaise, paresthesia in the upper limbs, and pain. Laboratory analysis revealed unremarkable findings in complete blood cell count, complete metabolic profile, and negative SARS-CoV-2 PCR test results.

Neurological examination revealed bilateral spasticity, strength 4/5 in the right limbs, and 3/5 and 2/5 proximal and distal, respectively, in the left upper and lower limbs. The muscle stretch reflexes were 3+ with a patellar clonus. An extensor plantar response was observed in both lower limbs. Exteroceptive hypoesthesia and hypopallesthesia were observed on the left side of the body. The sphincters were continent.

**Figure 1:**
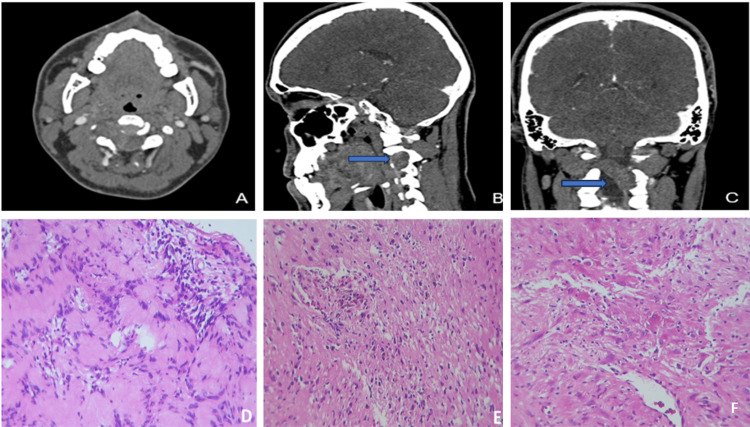
Neuroimaging and histology Contrast-enhanced computed tomography: axial (A), sagittal (B), and coronal (C) images show a well-rounded, lobulated, dense mass with inhomogeneous enhancement expanding the right neural foramen at levels C3-C4 (blue arrows). Histologically, the tumor showed Antoni A areas (D), focal Antoni B areas (E), and extended necrotic areas (F)(H&Ex400).

Magnetic resonance imaging (MRI) showed an extramedullary intradural lesion in C2-C4 that compresses the medullary canal. Contrast-enhanced computed tomography in the venous phase showed a well-rounded, lobulated, dense mass with inhomogeneous enhancement, expanding the right neural foramen at levels C3-C4 (Figures 1A-1C), suggestive of a schwannoma (SC). The patient was scheduled to undergo surgery in October. However, during this waiting period, the patient was vaccinated against COVID-19 (Moderna vaccine) and did not present subsequent symptoms.

**Figure 2:**
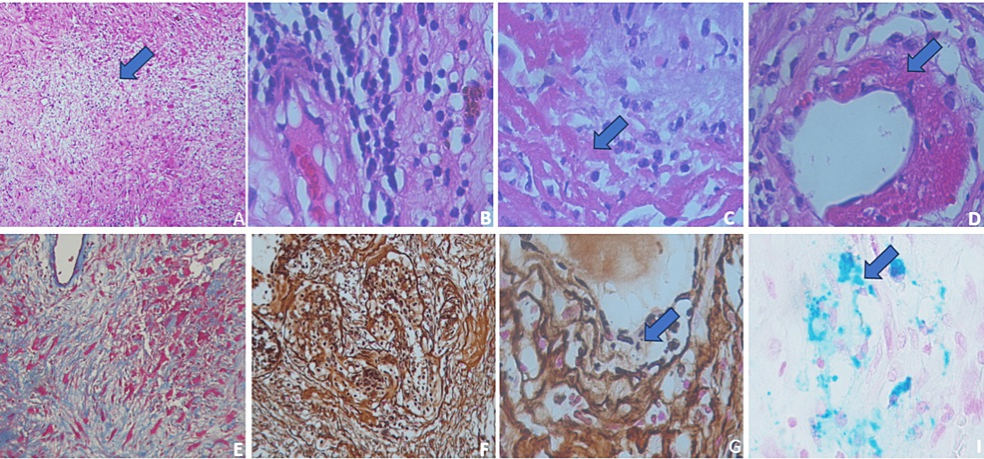
Histological findings and immunohistochemical analysis (A) Extended necrotic areas with regenerative changes (blue arrow) (H&Ex200). (B) Perivascular lymphocytic inflammatory infiltrate (H&Ex400). (C) Fibrinoid necrosis and inflammation affect the vessel wall. (D) Fibrinoid necrosis in the vessel wall (blue arrow) (H&Ex400). (E) Masson’s stain shows necrotic areas (x400). (F) Reticular fibers showed a disorganized patron with inflammation (x200) and (G) vessel walls with fiber fragmentation (blue arrow)(x400). (H) Macrophages with positive Perl’s stain (blue arrow) (x400).

The patient underwent surgery, and several tissue fragments measuring 20 × 10 mm were obtained. Reddish with soft yellowish cystic areas were found. Histologically, a schwannoma was identified with Antoni A areas (Figure 1D) and predominantly Antoni B areas (Figure 1E). Notably, the tumor showed zones of necrosis and loose areas (Figure 1F). With numerous newly formed blood vessels, vessels presented abundant lymphocytic inflammatory infiltrate of polymorphonuclear leukocytes (Figure 2A), which affected the vessel wall and endothelial cells (Figure 2B) as well as fibrin thrombi and extensive fibrinoid necrosis (Figure 2C-2D). Masson's staining revealed fibrosis in the area of necrosis (Figure 2E), and the reticular fibers showed fiber proliferation with a disorganized pattern (Figure 2F), as well as fiber fragmentation (Figure 2G). With Perl's staining, macrophages in blue suggested ancient hemorrhage (Figure 2H).

**Figure 3:**
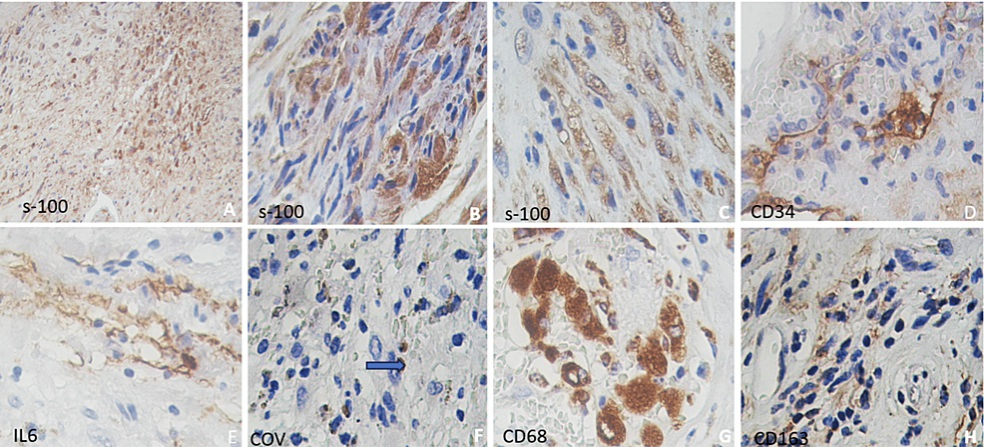
Immunohistochemistry (A-C) Postive for S-100. (D) Positive vessels for CD34. (E) Positive vessels and macrophages for IL-6. (F) Few positive macrophages for the SARS-CoV-2 antibody (blue arrow). (G) Abundant macrophages were positive for CD68+. (H) Focal positive expression for CD163+ (A-H) x400.

Immunohistochemistry showed positivity for vimentin and S-100 protein in the tumor (Figure 3A), while in the areas of necrosis, the Schwann cells showed strong positive immunoreactivity in the granular form (Figure 3B), and in other areas, extensive vacuolation was observed (Figure 3C). Endothelial cells showing edema and loss thereof, were positive for CD31, CD34 (Figure 3D), Factor VIII, IL6 (Figure 3E), IL10, PDGF, ACE2, and SARS-CoV-2 antibodies (Figure 3F). Few lymphocytes were mildly positive for CD3, CD4, and CD8, numerous macrophages were diffusely positive for CD68 (Figure 3G), and only a few showed CD163-positive immunoreactivity (Figure 3H). Based on the histological and immunohistochemical findings, the final diagnosis was schwannoma in C2 with fibrinoid necrosis and vasculitis probably because of COVID-19 infection or secondary changes to the vaccine. The patient experienced no postoperative complications or sequelae. Follow-up was performed with surveillance in the outpatient clinic and semi-annual observation with a neuroimaging study.

## Discussion

We report the case of a young patient who exhibited prolonged fever, diarrhea, and substantial weight loss. Although COVID-19 testing was not conducted after the initial improvement, COVID-19 was considered. Subsequently, the patient developed arm paresthesia and pain, leading to an MRI that revealed a C2 lesion diagnosed as a SC. One week before the surgery, the patient was vaccinated against COVID-19 using the Moderna vaccine. Biopsy showed vascular hyalinization, mucoid deposition, cyst formation, increased blood vessels, fibrinoid necrosis of vessels, necrosis, inflammation, macrophages, hemosiderophages, hyalinization vessels, and endothelial cell damage.

Here, we have several questions that we asked ourselves, and the secondary histological features correspond to:

1. These are secondary changes of chronic COVID-19 with six months of evolution in an asymptomatic patient with a recent negative PCR test.

2. These correspond to post-vaccine changes affecting a COVID-negative patient's tumor.

3. They correspond to degenerative changes described in SCs, or it is a coinfection in an existing tumor.

4. These are in addition to cofactors such as post-COVID-19 immunoexpression or patients with immunosuppression by cocaine and alcohol consumers, as well as a post-vaccination change or corresponding to an infected SC, as they have been reported to be associated with bacterial infections [7].

5. It is a secondary vaccine change.

SC was diagnosed based on classic Antoni A and B patterns. Interestingly, in similar cases, degenerative alterations were noted in the Antoni B area, characterized by nuclear and cytoplasmic vacuolation, absence of mitotic forms, and loss of S-100 immunoreactivity. These changes may be attributed to the random organization of cells [1]. Much like the observations made by Abe et al., we found that the proliferating index (measured by Ki67 or MIB-1) was elevated at the borderline region between Antoni type A and B patterns, accompanied by a substantial infiltration of macrophages (CD68+) in contrast to the remainder of the tumor [10].

Ancient SCs are often linked to secondary degenerative alterations, such as the accumulation of mucoid material, the development of cysts, a prominent upsurge in blood vessels, subsequent hemorrhage, and the presence of numerous macrophages containing hemosiderin, along with dystrophic calcification [2, 3]. As mentioned, these alterations were observed in our case.

The inflammatory component in the pathogenesis of this tumor highlights the role of macrophages, associated with the elevation of their proliferation to repair and help in the phases of damaged tissues. Macrophages are classified into M1 (CD68+) and M2 (CD163+). M2-type macrophages have been associated with tumorigenesis, angiogenesis, and suppression of antitumor immune responses, thereby supporting cancer cell survival [11-13].

Specifically, in the peripheral nervous system, macrophages play an important role in immune cells. Several studies have shown that macrophages multitasked by removing myelin debris together with Schwann cells also promote axonal regeneration through the liberation of extracellular matrix (ECM) proteins, growth factors, cytokines, and chemokines [11, 12]. Secondary to these changes, it is possible to observe by immunohistochemistry different phases of nerve regeneration and Wallerian degradation, myelin fragmentation, and the predominance of macrophages and lymphocytes [12]. As part of the presentation of our case, the predominance of immune cells, lymphocytes, and polymorphonuclear cells could be associated with an acute bacterial infection process, as in the case of Kishi et al. [7]. Intratumoral necrosis has not been previously reported in degenerative changes or ancient SCs, as in our case. This finding could be associated with malignant transformation or tumor progression [1-2, 14]. Hemorrhage may be related to degenerative changes and the presence of hemosiderophages and does not indicate vascular changes or chronic hemorrhage. Few cases of intracerebellar hemorrhage associated with COVID-19 vaccination have been reported, and these have been identified as vasculitis, endothelial injuries, and thrombosis [15-17]. They want to make a special mention of the cases reported by Sugasawa et al. [16]. We reported two cases of brain tumors with intratumoral hemorrhage as a likely lateral effect of COVID-19 mediated by the vaccine. In one case of vestibular SC and a second case of meningioma, none had risk factors for bleeding. In the case of SC, intratumoral hemorrhage, an abundance of foamy macrophages, and adjacent necrosis [16]. The histological findings described in this case report were similar. Our patient also presented with a vascular injury.

A possible comprehension of endothelial damage would be the interaction of SARS-CoV-2 and ACE2. ACE2 participates in the renin-angiotensin-aldosterone system by regulating angiotensin II conversion. However, endothelial cells and other tissues predominate in the endothelial membrane and are the entry receptors for SARS-CoV-2. The systemic inflammation of the infection and endothelial damage leads to abnormal prothrombotic changes, producing vasculopathy. Moreover, the acute immune response by the microglia, considered as resident populations of macrophages, to the infection activates the NLR family pyrin domain-containing 3 (NLPR3, also known as NACHT, LRR, and PYD domain-containing protein 3) inflammasome by the secretion of TNF-α and IL-1β. Similar cases of infection with the Zika virus and Japanese Encephalitis virus lead to cell damage and death [18,19]. This suggests that endothelial effects could be present prior to vaccination, possibly because of a previous SARS-CoV-2 infection.

## Conclusions

We report a complex case involving a schwannoma with immune responses, degenerative changes, and intratumoral necrosis, with potential COVID-19 infection and vaccination. These histological findings raise intriguing questions about the origins of the observed alterations, with several potential explanations. Understanding the relationships between these factors and their implications for patient care and outcomes requires further research. Additionally, the involvement of macrophages, immune responses, and endothelial damage in the pathogenesis of tumors adds complexity to the case, warranting in-depth exploration. Ultimately, this case underscores the importance of a multidisciplinary approach and ongoing research to elucidate the underlying mechanisms and optimize patient management.
